# Lifestyle and Depression among Hong Kong Nurses

**DOI:** 10.3390/ijerph13010135

**Published:** 2016-01-16

**Authors:** Teris Cheung, Paul S.F. Yip

**Affiliations:** 1 School of Nursing, Hong Kong Polytechnic University, Hong Kong, China; 2 Centre for Suicide Research and Prevention, University of Hong Kong, Hong Kong, China; sfpyip@hku.hk

**Keywords:** DASS-21, depression, epidemiology, nurses, therapeutic lifestyle changes

## Abstract

Recent longitudinal data suggest a close association between depression and lifestyle. Little work to date has estimated the prevalence of depression in the nursing workforce in China, nor considered what lifestyle factors might be correlated with it—a gap filled by the present study. The study’s web-based cross-sectional survey solicited data from qualified nurses aged between 21 and 65 registered with the Hong Kong Nursing Council. The Depression, Anxiety and Stress Scale 21 was used to measure 850 nurses for depression, anxiety and symptoms of stress; a generalized linear regression model examined associations between lifestyle factors and depression. Mean depression symptom scores show a downward linear trend for male and female participants. Gender and age, however, did not emerge as significant predictors of depression. Three lifestyles factors (sleep, entertainment and hobbies) showed a significant association with depression. Nurses should make therapeutic lifestyle changes to improve their work-life balance and safeguard their functioning at work and personal well-being.

## 1. Introduction

It is clearly worthwhile for scholarship to investigate the correlation of neuropathological disorders like depression—or indeed of psychological states like happiness—with lifestyle factors; yet to do this, there is a need first for a workable definition of “lifestyle”. Three decades ago, lifestyle was defined as “any distinctive and recognizable mode of living” [1]. We can also understand the term as comprising the key elements making up an individual’s health and well-being. Swarbrick [2] defines another term, “wellness”, as “a conscious, deliberate process that requires a person to become aware of and make choices that help promote a more satisfying lifestyle”. Well people, for Swarbrick, have healthy, balanced habits. They sleep adequately; they eat well; they exercise; they are productive at work and have meaningful leisure pursuits and social relationships. These things keep them in good physical and mental health. People who fail to maintain a healthy lifestyle can come to suffer from physical disease and risk their balance of mind [3].

It is often nurses who encourage patients to change their behavior in the sense of adopting a healthier lifestyle [4]. The clinical effectiveness of these interventions has been proven. So far, there have been few studies of ethnically Chinese nurses’ psychological well-being [5], especially in connection to lifestyle. Interestingly, interventions into patients’ lifestyles have rarely been tested on nurses themselves [6]. With the HKSAR population growing by over 7 million since 2014, nurses have to deal with the increasingly complex care of greater numbers of patients in hospitals. Unfortunately, nursing in Hong Kong tends to be short-staffed and badly remunerated [7,8]. Nurses, further, often work under serious time pressure with inadequate resources and fractious colleagues [9], and can sometimes encounter workplace violence [10,11]. With advances in medical technology, the public may have higher expectations of nurses’ professional behavior, putting nurses at higher risk of developing occupational stress and associated forms of psychiatric morbidity [12,13,14,15].

In this circumstances, it is unsurprising that nurses may have failed to develop effective stress-coping mechanisms; they may, in fact, take greater lifestyle risks with their health than the population at large [16,17]. Existing research on lifestyle mostly focuses on patients. The empirical evidence for nurses’ lifestyles over the last decade is scant. This means little is known about the impact of unhealthy lifestyles on the working-age nursing population. There is a need for a serious prevalence estimate of depression in nurses—otherwise cultural assumptions may jeopardize nurses’ mental health or compromise their ability to seek timely professional or psychiatric help.

The World Health Organization (WHO) estimates that, by 2030, depression will become one of the three leading causes of disease burden globally [18]. The WHO also project that by 2020, the same disorder will be the second leading cause of poor health and mortality [19]. Depression is one of the closest correlates with suicide in China [20], and suicide is itself among the top five causes of death in all nurses, from the newly qualified to retirees [20]. In several countries, female nurses feature among the occupational groups identified as being at elevated risk of suicide [21,22,23,24]. This risk may reflect occupational stresses including heavy workload, lack of autonomy and job dissatisfaction [25]. Recent research has also underscored how shift work is a major factor causing stress and burnout among nurses [26]—two factors closely linked to depression. More significantly, nursing is predominantly a female profession; depression is further more prevalent in women [27]. The current study investigates possible linkages between lifestyle and depression in nurses. Recent research [28] outlines causal relationships between lifestyle and depression in the general population. A Japanese longitudinal study of 9201 individuals, aged between 40 and 69, uncovered associations between physical inactivity, poor self-perceived health, chronic illness and depression [28]. The longitudinal study showed that participants of both sexes with poor self-perceived health and chronic illness were more likely to become depressed. Multivariate analyses confirmed this increased risk of depression in male participants who were physically inactive and had chronic disease, and in female participants with a body mass index of 25 or more and poor self-perceived health. In other words, elements of both lifestyle and health status emerged as risk factors for depression [28].

As far as we know, no local study has investigated associations between lifestyle and depression among nursing professionals in Hong Kong. While some health professionals may have healthy lifestyles in terms, say, of nutrition, they may underestimate how this can be undermined by unhealthy habits like not exercising or sleeping well. This paper studies the multiple psychopathology of depression by reporting statistical relationships between lifestyle factors and depression, stressing a holistic conception of health as the basis for maintaining nurses’ mental health.

## 2. Experimental Section

### 2.1. Aims

This study sets to answer the following questions: (1) Is there any gender difference in depression among Hong Kong nurses? (2) Is there any association between lifestyle factors and depression? (3) What significant lifestyle factors are associated with depression among nurses?

### 2.2. Study Design

This study used a cross-sectional survey. It took account of existing nursing literature on mental health in designing a web-based survey, administered by nurses to themselves. This paper only reports weighted prevalence estimates for depression as measured by a short version of Depression Anxiety and Stress Scale (DASS 21) [29], together with mental health-related components.

### 2.3. Participants

The study’s sampling frame drew on a database maintained by the Association of the Hong Kong Nursing Staff (AHKNS), the fullest, most current registry of contact information for practitioners in the territory. The Association’s 24,000 registered nurses comprise over 50% of the nurses on the books of the Hong Kong Nursing Council. Nurses joined the Association on a voluntary basis. A total of 67% (16,082) of the Association’s members have contact details with the body. The Association sent out a mass email inviting participation in our anonymous self-reported web-based survey. To count, nurses had to be: (1) qualified with the Hong Kong Nursing Council, HKSAR; (2) aged between 21 and 65 irrespective of gender. We excluded non-readers of Chinese as our survey was in this language. Data collection period spanned a 4-week period from October to November 2013.

### 2.4. Ethical Considerations

The study was approved as a social science project by the Human Research Ethics Committee for Non-Clinical Faculties (HRECNCF) (Reference No: EA 030813) and the Institutional Review Board of a local university in Hong Kong. Prior written informed consent was obtained from all participants. Since some questionnaire items were sensitive, emergency helplines were listed on the survey’s last page.

### 2.5. Data Collection Tools and Measurements

The survey comprises nine sections and takes approximately 20 min to complete. It elicits socio-demographic and other work-related information through respondents’ self-administered responses. Depression, anxiety and symptoms of stress are measured by a short version of the Depression Anxiety Stress Scale (DASS) [29].

#### 2.5.1. Instrument

##### Depression Anxiety Stress Scale 21 (DASS-21)

The Depression and Anxiety Stress Scale 21 (DASS_21_) is a validated and reliable self-report 4-point Likert scale measuring three dimensions of mental health: depression, anxiety, and stress [29,30,31]. The instrument is frequently used in clinical [32] and non-clinical samples [29,31,33,34] and Asian countries, across age groups [6,34], possessing well-established psychometric properties in reliably measuring depression, anxiety and stress (at a Cronbach’s alpha 0.91, 0.84, and 0.90 respectively). It is also believed capable of differentiating between depression, anxiety and stress [29,34].

DASS 21 items are scored on a 4-point scale ranging from 0 (“did not apply to me at all”) to 3 (“applied to me very much”). Three subscales have seven items: Depression (example item: “I couldn’t experience any positive feeling at all”), Anxiety (e.g., “I found it hard to breathe”), and Stress (e.g., “I found it hard to wind down”). Higher scores indicate more severe symptomatology. In the study, scores from each dimension were summed up and categorized as “normal”, “mild”, “moderate”, “severe”, and “extremely severe”. The DASS severity ratings were shown in Table 1 below [29].

**Table 1:** DASS Severity Ratings.

Severity	Depression	Anxiety	Stress
Normal	0–9	0–7	0–14
Mild	10–13	8–9	15–18
Moderate	14–20	10–14	19–25
Severe	21–27	15–19	26–33
Extremely Severe	28+	20+	34+

Participants with a cut-off score of ≥10 in the depression dimension were taken as depressed in the terms of the DASS manual [29].

##### Lifestyle

The survey posed six questions relating to lifestyle habits to measure how often respondents adopted positive lifestyle habits as expressed using a 5-point Likert scale (0: Never; 1: Rarely; 2: Occasionally; 3: Always; 4: All the time). These single-item questions were derived from our experience with interventions promoting better health and with the stress management workshops attended by nurses. Single-item measures of lifestyle can be inherently valid in reflecting respondents’ own perceptions of lifestyle [35]. This set of questions was distributed to a panel of mental health experts comprising five members (a Professor in Mental Health Nursing; an Assistant Professor and a Senior Clinical Associate in the same field and two Charge Nurses working with Mental Health). Face and content validity were approved by all the experts before the survey started collecting data. The survey classifies activities on the basis of a review of the existing literature [3,36]. We categorized lifestyle activities into five domains (1: dietary habits; 2: sleep pattern; 3: physical activity levels; 4: social/recreational activities; 5: activity and passivity).

Respondents were asked to report their frequency of participation in the following activities specific to a usual week. The lifestyle questions asked were as follows:

1. I ate at least one hot, balanced meal a day (Domain 1: dietary habits)
2. I slept 7–8 h for at least 4 nights in a usual week (Domain 2: sleep pattern)
3. I exercised (to the point of sweating) at least twice a week (Domain 3: physical activity levels)
4. I found time for entertainment at least once a week (Domain 4: social/recreational activities)
5. I kept up hobbies (like gardening or playing music) once a week (Domain 4: social/recreational activities)
6. I had some time to myself every day (Domain 5: activity and passivity)

### 2.6. Statistical Analysis

Statistical analysis was conducted in SPSS 23.0 (SPSS Inc., Chicago, IL, USA). Raw data were weighted and adjusted before estimating prevalence, with independent-sample t-tests and chi-square tests used to examine mean differences in depression symptom scores between male and female participants, those with or without shift work, and those satisfied or dissatisfied with their job. Differences were taken as significant at 0.05 level (2-tailed). Lifestyle factors were analyzed in the generalized linear regression model. Gender, age and shift work were controlled as confounding variables.

## 3. Results and Discussion

A total of 850 participants (female = 745) completed the web-based survey, at a response rate of 5.3%.

### 3.1. Socio-Demographic, Clinical and Other Characteristics of the Sample Population

Most respondents were female (87.6%, *n* = 745), front-line nurses (87.2%, *n* = 740) aged between 25 and 34 years (*SD* ± 2.79). 43% were single, 55% married and 2% divorced, separated or widowed. Female nurses showed a higher mean depression score (8.37 ± 8.71) than male (8.27 ± 9.51). Nearly 70% (*n* = 601) held a bachelor’s-level degree or above and earned (part of) a monthly household income of HKD 40,000–59,000. Participants (in general and psychiatric streams) had an average of 10 to 20 years’ clinical experience. Approximately 70% (*n* = 603) worked in a shift rotation. 64% (*n* = 543) expressed satisfaction with their jobs. Most respondents were non-smokers (98.5%). About 24% of participants had drunk alcohol in the last month. 19.1% called themselves occasional drinkers, 4.7% habitual drinkers. Shift workers (Mean = 8.77, *SD =* 9.05) had a significantly higher mean depression symptom score than those non-shift workers (Mean = 7.36, *SD =* 8.11). (F = 2.85, *df* = 848, *p* < 0.05, 95% CI −2.71, −0.11). Gender, age, shift work and lifestyle factors were primarily our variables of interest in examining the association with depression in nurses.

#### Prevalence Estimates of Depressive Symptoms by Gender and Age

Table 2 reports the weighted prevalence of past-week depression by gender and age. Depressive symptoms were more prevalent in female nurses (36.6%, 95% CI 36.01–37.19) than their male counterparts (30.3%, 95% CI 29.66–30.94). Age and depression relate according to a downward linear trend: younger nurses tend to report more depressive symptoms than older nurses.

**Table 2:** Weighted prevalence (95% CI) of past-week depression by gender and age.

	*N* (850)	Prevalence % (95% CI)
Gender		
Male	105	30.3 (29.7–30.9)
Female	745	36.6 (36.0–37.2)
Age (years)		
21–24	77	39.3 (39.2–39.4)
25–34	275	38.1 (38.0–38.2)
35–44	283	37.8 (37.7–37.9)
45–54	186	31.0 (30.9–31.1)
55–64	29	17.3 (17.3–17.3)
Total	850	35.8 (35.7–35.9)

Table 3 reports nurses’ distribution of depression scores by severity. The overall prevalence of depression is 35.8%. 12.2% of nurses report mild depressive symptoms; 23.6% report moderate to extremely severe symptoms.

**Table 3:** Severity distribution (%) of Depression scores among nurses in the present survey.

Variable	*N*	Normal	Mild	Moderate	Severe	Extremely Severe
Depression	All (*n* = 850)	64.2 (*n* = 545)	12.2 (*n* = 104)	15.0 (*n* = 128)	3.2 (*n* = 27)	5.4 (*n* = 46)
	Male (*n* = 105)	69.7 (*n* = 73)	6.7 (*n* = 7)	11.8 (*n* = 12)	5.1 (*n* = 5)	6.7 (*n* = 7)
	Female (*n* = 745)	63.4 (*n* = 472)	12.9 (*n* = 96)	15.5 (*n* = 116)	3.0 (*n* = 22)	5.2 (*n* = 39)

Table 4 reports the bivariate correlations between depression scores, sex, age, shift duty and lifestyle factors. All lifestyle factors were significantly correlated with depression (all *p*s < 0.05, 2-tailed). Gender and shift work were not significantly correlated with depression scores. Age seemed to be a significant correlate of depression (*r* = −0.083, *p* < 0.05, 2-tailed).

Table 5 reports results from the Generalized Linear Model. Three lifestyle factors (1: poor sleep; 2: not taking time out for entertainment; and 3: not keeping up hobbies) were significantly associated with depression (all *p*s < 0.05), with an intercept (ß) of 2.610, SE = 0.179, *p* < 0.001, 95% CI 2.26 to 2.96. The adjusted R squared is 0.083 indicating that 8.3% of the variance in depression is explicable through the three significant lifestyle factors. Gender, age and shift work were not found to be significant correlates of depression.

**Table 4:** Correlations (Pearson’s rho) between DASS depression scores, gender, age, shift work and lifestyle factors.

	DASS Depression Score	Gender	Age	Shift Work	Hot and Balanced Meal	Sleep	Exercise	Entertainment	Hobbies	Quiet Time
**DASS depression score**	1									
**Gender**	0.025	1								
**Age**	−0.083 *	0.052	1							
**Shift Work**	0.050	−0.031	−0.244 **	1						
**Hot and balanced meal**	−0.180 **	−0.057	0.114 **	−0.173 **	1					
**Sleep**	−0.273 **	−0.003	0.171 **	−0.235 **	0.410 **	1				
**Exercise**	−0.196 *	−0.155 **	0.180 **	−0.110 **	0.287 **	0.299 **	1			
**Entertainment**	−0.267 **	−0.087 *	0.066	−0.105 **	0.375 **	0.406 **	0.429 **	1		
**Hobbies**	−0.275 **	−0.093 **	0.154 **	−0.150 **	0.326 **	0.362 **	0.503 **	0.506 **	1	
**Quiet time**	−0.195 **	−0.021	0.142 **	−0.066	0.314 **	0.378 **	0.313 **	−0.150 **	0.506 **	1

Notes: * Correlation is significant at the 0.05 level (2-tailed). ** Correlation is significant at the 0.01 level (2-tailed).

**Table 5:** Generalized linear model, parameter estimates, dependent variable: DASS depression score.

Parameter	B	Std. Error	*t*	Sig.	95% CI Bound		Partial Eta Squared
					Lower	Upper	
**Intercept**	2.610	0.179	14.55	0.000	2.26	2.96	0.201
**Gender**							
**Female**	−0.006	0.069	−0.084	0.933	−0.14	0.13	0.000
**Male ^#^**	-	-	-	-	-	-	-
**Age (years)**							
**21–34 ^#^**	-	-	-	-	-	-	-
**35–44**	0.080	0.073	1.088	0.277	−0.06	0.22	0.001
**45–64**	0.093	0.074	1.263	0.207	−0.05	0.24	0.002
**Shift Work**	−0.047	0.064	−0.736	0.462	−0.17	0.08	0.001
**No Shift Work ^#^**	-	-	-	-	-	-	-
**Hot and balanced meal**	−0.013	0.035	−0.370	0.712	−0.08	0.06	0.000
**Sleep**	−0.127	0.030	−4.266	0.000	−0.18	−0.07	0.021
**Exercise**	−0.026	0.031	−0.856	0.392	−0.09	0.03	0.001
**Entertainment**	−0.091	0.040	−2.278	0.023	−0.17	−0.01	0.006
**Hobbies**	−0.094	0.036	−2.633	0.009	−0.16	−0.02	0.008
**Quiet Time**	0.000	0.036	0.005	0.996	−0.07	0.07	0.000

Notes: ^#^ Reference category. Computed using alpha = 0.05 (2-tailed). R squared = 0.096 (Adjusted R Squared = 0.083). Gender, age and shift duty were controlled confounding variables.

### 3.2. Discussion

Our prevalence rate of depression (35.8%) is significantly lower than that found in another recent study (*n* = 1592) examining the prevalence of depression (and associated factors) among Chinese nurses [5]. Gao finds a prevalence of 61.7% (*n* = 886), with 74.9% of respondents (*n* = 664) having mild depressive symptoms—six times higher than our figure. In the United States, a similar study [37] reveals a 35% (*n* = 150) prevalence rate of female nurses suffering from mild to moderate depressive symptoms. Our overall prevalence rate of mild to extremely severe depressive symptoms is again lower than these findings.

Research consistently finds a higher prevalence of depression in women than men. Park *et al.* [38] examine associations between job-related stress and depressive symptoms in 3103 employees in small-and-medium sized enterprises in Korea using the CES-D from September 2006 to March 2007. Female employees exhibit more depressive symptoms than men, at rates of 26.1% male to 28.7% female. Although female nurses reported depression symptoms more often than their male counterparts in our study, results from our bivariate correlational analysis and the Generalized Linear Model suggested that gender was not a significant correlate in depressive symptoms (*r* = 0.05, *p* = 0.12, 2-tailed), making our findings inconsistent with Park’s. In the past few decades, research has revealed that features associated with certain jobs (like strain and burnout) may have profound effects on employees’ physical and mental well-being [25]. Nursing is characterized by high time-pressure and low autonomy—a combination that can induce psychological stress and illness. Unclear about their responsibilities or the latitude they may exercise in taking decisions, some nurses may become exhausted and develop sleeping problems, imperiling their physical and mental health [39]. Male and female nurses in Hong Kong may have equal exposure to these work-related stresses, which may further develop into chronic stress and at worst depression [40].

Participants unable to sleep 7–8 h 4 nights a week were more likely to become depressed. It is unclear whether sleep deprivation is always a consequence of nurses’ shift rotations. Our results showed that respondents requiring shift work rotations had higher mean score of depression (Mean: 8.77, *SD =* 9.05) than those on regular day shifts (Mean: 7.35, *SD =* 8.11). Nevertheless, our bivariate correlational analysis and the Generalized Linear Model showed that shift work was not significantly associated with depression (*r* = 0.05, *p* > 0.05 (2-tailed) and *p* = 0.46, 95% CI −0.17, −0.08 respectively). There were three speculations why shift work did not come out as significant correlates of depression in the bivariate correlations and GLM. First, approximately 70% of nurses required shift work in this study, of which 43% were single. Thus, the disruptions of family routine and functional roles caused to single nurses may be less severe compared to those married participants. Second, our sample was relatively young; 75% (*n* = 635) of participants were between 21 to 44. Younger nurses may not have yet experienced the long term negative physical and psychological impact brought about by shift work. Third, some nurses may actually enjoy shift work as they have more time off in the weekdays to perform their functional roles.

Besides, sleep deprivation and disturbance are different constructs: the first is subjective, while the second can be measured objectively (e.g., by polysomnography). Prolonged sleep deprivation may lead to sleep disturbance, which has been taken as a key symptom of depression in psychiatry [41,42]. Over 70% of respondents worked in shifts. It would be possible to argue that depression and sleep disturbance represent mutual causes and effects—more depressed nurses request anti-social shifts, while night-work brings about depression. Sleep disturbance can affect nurses’ job performance [26] and thus the quality of patient care.

Shift work rotation would seem inevitably to cause de-synchronization in circadian rhythms, in extreme cases inducing disturbance [43]. This, in turn, affects formal and informal social participation, family relations and engagement in solitary activities (e.g., entertainment and hobbies) [44]. Shift work may disrupt nurses’ family routines and functional roles, as rotations place them on a different schedule from their families. Shift work may also interfere with nurses’ relationship with their spouses and children. Examining the effect of shift work on nurses’ physical and mental health, Skippers *et al.*’s [45] survey of 463 American nurses found no significant relationship between nurses’ physical and mental health. Shift work, though, correlated strongly with sleep quality and quantity (*p* < 0.001). Admi *et al.* [44] also find no basis for considering shift work as a risk factor for nurses’ health and organizational outcomes. Older research like Jarnal & Jarnal [46] found that nurses on rotating shifts scored higher for depression than other nurses. Jarnal’s findings consistently indicate that shift work does exert a negative impact on nurses’ mental health, in line with Stimpfel *et al.*’s [26] claims. Two recent reviews further find that a reduction in shift work rotations improves patient outcomes, patient safety and patients’ quality of life [47,48].

Whatever the disagreements between researchers about shift work’s psychological effects, our findings showed that shift work rotation has exerted some impact on nurses’ mental health. In Hong Kong, nurses were required to have 4 to 5 night shifts per calendar month, with an average of 1 to 2 nights per week. In this regard, shift work has certainly caused physical exhaustion and sleep disruptions to nurses, albeit in different extent. Thus, the Hong Kong health authority could usefully investigate associations between shift-work rotations and mental health among nurses.

Consistent with other research [3,49], we found nurses not being able to keep up hobbies significantly correlated with depression. Bivariate analysis showed that keeping up hobbies and finding time for entertainment were significantly associated (*r* = 0.64, *p* < 0.001 (two-tailed)). Outside interests are vital in physical and mental health [49]. Hirosaki *et al.*’s [49] cross-sectional study of 658 older residents of a Japanese community compared their activity of daily living (ADL), depression, and quality of life scores. Results indicate that respondents with hobbies (*n* = 372) had significantly higher quality of life scores, lower depression scores and a higher ADL score (all *p*s < 0.001). Comparably, Parisi *et al.’s* [3] longitudinal study of 328 women in Baltimore (MD, USA) looked at the effects of baseline activity on depressive symptoms over a 6-year period. Discrete-time Cox proportional hazards models measure indicative depressive symptoms in four activity domains (1: intellectual; 2: creative; 3: social; 4: passive). Results showed that frequent engagement (*i.e.*, 2–3 times per week) in creative activity (e.g., playing an instrument, sewing, drawing, painting) was associated with a 7% decrease in the risk of depressive symptoms (HR 0.92, 95% CI 0.87, 0.98). No statistically significant association obtained between the three other activity domains and depression.

Since Hirosaki’s and Parisi’s studies were concerned with older people, their results may not match ours in that Hong Kong nurses of working age could have different expectations regarding lifestyle and quality of life. Nevertheless, recent research has stressed that leisure activities arouse positive emotions, promote self-efficacy, increase competency, and act as buffers for stress [50,51]. Maintaining an active lifestyle was associated with lower levels of depressive symptoms [52,53]. Given the frustrations of their job, nurses may be especially in need these forms of recreation and relaxation. Through hobbies and entertainment, nurses may regain a sense of mastery and self-control, boost their self-esteem, reinforce their relationships and experience periods of happiness before they return to work [3,54]. Some research suggests some individuals can positively affect their well-being through making enlightened lifestyle choices [54]. The social and psychological benefits gained from participation in a variety of activities may also help reduce social isolation as this leads to depression [50]. These considerations may explain why keeping up hobbies and finding time for entertainment jointly stood out as significant lifestyle factors in the regression model.

## 4. Implications of This Study

This article sets out the statistical relationship between nurses’ lifestyle and depressive symptoms. Sleeping well (7–8 h sleep at least 4 times per week) and keeping up hobbies and some form of relaxation or entertainment (at least once per week) was significantly linked with less depression even though the causality between these factors cannot be established. Although other lifestyle factors investigated by the study (e.g., a balanced diet, exercise, quiet time) did not turn out to be statistically significant for depression, this does not mean they do not deserve further attention. The study rather invites replication through longitudinal research or in-depth focus group interviews further examining casual linkages between lifestyle factors and depression in Hong Kong nurses.

The central theme of this study is “lifestyle choices”. Findings emerged from this study suggesting that certain lifestyle factors were significantly associated with depression among Hong Kong nurses. Our study findings were consistent with those of other authors [3] who undertook similar research on the general population. While some lifestyle factors were found to be significantly associated with self-perceived depressive symptoms, all factors are amenable to change. In theory, individuals can themselves control their health and lifestyle [36]. Efforts in health promotion should seek to empower individuals to make healthy lifestyle choices preventing chronic disease, premature morbidity and death, and reducing the occurrence of psychiatric morbidity.

## 5. Limitations of the Study

The study is limited by its reliance on self-reported measures of lifestyle factors and depressive symptoms, which were anyway confined to a usual week before the study period. Objective measurement of depressive symptoms (e.g., through clinical interviews) may yield different results. Nevertheless, our findings remain clinically relevant since self-reported depressive symptoms influence health and can indicate the future onset of depression [27]. The composition of our survey with self-selecting participants may make it difficult to extrapolate statistical findings to the general population. The response rate of 5.3% was arguably too low to generalize findings to the nursing population. Additionally, the study’s cross-sectional design can only describe statistical relationships between lifestyle factors and depression. A randomized control design or other experimental approach would be necessary to draw causal conclusions. A longitudinal questionnaire study may, however, be a first important step in this direction.

## 6. Conclusions

This paper examines the statistical relationship between lifestyle and depression. Three lifestyle factors (failure to sleep 7–8 h 4 nights a week; failure to keep up hobbies and to find time out for entertainment) were associated with lower rates of depression. The study examines how lifestyle choices were related to self-rated depression in the nursing profession. Depression is demotivating and can make it hard for sufferers to remain active or make healthier lifestyle choices. The implication is that strategically-focused interventions on mental health problems may lead to improvement in nurses’ lifestyle choices and vice versa.
